# Moral jeopardy, conflicts of interest and the integrity of public health research

**DOI:** 10.1093/ije/dyae023

**Published:** 2024-02-19

**Authors:** Peter J Adams, Melissa-Jade Gregan

**Affiliations:** Centre for Addiction Research, School of Population Health, The University of Auckland, Auckland, Aotearoa New Zealand; Centre for Addiction Research, School of Population Health, The University of Auckland, Auckland, Aotearoa New Zealand

**Keywords:** Unhealthy commodities, commercial determinants, industry influence, conflicts of interest, alcohol, tobacco, food, gambling

Moral jeopardy occurs when researchers or research organizations committed to public good opt to accept profits from industries in ways that generate conflicts of interest. The United States Institute of Medicine defines conflicts of interest as ‘circumstances that create a risk that professional judgments or actions regarding a primary interest will be unduly influenced by a secondary interest’.^1^ Primary interests include protecting the integrity of research and secondary interests include not only financial gains, but more subtle advantages such as improved connections or professional standing. Conflicts of interest need to be taken seriously because they not only impact on the perceived independence of a piece of research, but also jeopardize the researchers’ future reputation and academic integrity.^2^ For example, a health research institute that accepts funds directly from a wine producer is likely to be perceived, given the health impacts of alcohol, as morally duplicitous and, on further receipt of such funding, their reliance on this source could be seen as compromising their academic independence. In academic contexts in which funding sources are poorly monitored, such relationships can pass by unnoticed and inconvenient questions of morality can be easily dismissed by using available justifications to silence any nagging doubts. For example, during 2018, Public Health England accepted funding from an alcohol industry front group, Drinkaware, to fund their Drink Free Days campaign on the basis that it enabled them to take an active role in reducing alcohol-related harm.^3^ However, brushing such concerns aside could entail ignoring some areas of risk that may have serious consequences at a later stage.

Public health researchers have an important contribution to make to healthy public policy, but research evidence on effective measures is often ignored in favour of less effective measures. For example, over the last 20 years, policymakers attempting to reduce alcohol-related harm have favoured less effective individualized approaches, such as alcohol education and awareness funded and/or developed by the alcohol industry itself, over more effective approaches such as changes in price and availability.^4^ A key factor in industry success in diverting or blocking effective policy has been the strategies aimed at influencing and distorting the knowledge base. In other words, industries invest in ongoing and innovative ways to shape how public health research is conducted and the topics researchers choose to pursue.^5^^,^^6^

## Commercial determinants of health

The study of the commercial determinants of health and industry political influence is a relatively new but rapidly expanding subfield of public health.^7^ Its newness has posed challenges in finding a common understanding of key concepts and processes.^8^ For example, the World Health Organization refers to this field of study as ‘commercial determinants of health’;^9^ other labels include ‘corporatology’,^10^ ‘dangerous consumptions’,^11^ ‘addiction industry studies’^12^ and ‘unhealthy commodity industries’.^13^ These industries can be divided roughly into three categories: (A, B and C: Addictive, Behaviourally challenging and Contextually damaging).^14^ Category A (‘Addictive’) commodities, such as tobacco, alcohol and gambling, need to be treated differently from other unhealthy commodities primarily because addicted consumers differ from non-addicted consumers in several important ways: addicted consumers consume far more than non-addicted consumers and are, therefore, responsible for increasing the scale of the profits, and this provides a strong incentive for producers and retailers to oppose legislative measures that might curb such consumption.^15^^,^^16^

Category B (‘Behaviourally challenging’) commodities, such as ultra-processed food, guns, pornography and cosmetic surgery, miss out on the addiction-inflated profits but it is still commonplace for these industries to advance their interests using similar tactics such as manipulating research. For example, the nutrition academic Marion Nestle has documented conflicts of interest that nutrition researchers have encountered with food industry funding.^17^ This includes funds accepted by nutrition research institutes, nutrition societies, nutrition conferences as well as journals publishing nutrition research.

Category C (‘Contextually damaging’) commodities, such as military armaments, fossil fuels, commercial logging and nuclear power, focus more on consumptions by collectives of people. Although they too miss out on the dynamics of addiction-inflated profits, they are exposed to the power play of large multinationals, and that, unsurprisingly, leads to a similar use of influence strategies as occurs with categories A and B, such as undermining the knowledge base.^18^ Indeed, the three categories are not mutually exclusive and considerable overlaps occur both in terms of form and tactics. For example, pharmaceutical industries include products that are addictive (such as benzodiazepines) and some corporations trade in more than one commodity (such as supermarkets selling both alcohol and tobacco).

## Risks to consider

Studies that investigate industry tactics are uncovering increasing evidence of their influence on public health research and researcher attempts to disguise these connections. For example, Philip Morris International has, under the banner of their Foundation for a Smoke Free World, funded a range of initiatives, including research, with a view to accelerating the uptake of vaping.^19^ The counterpart here is researcher willingness to buy into industry initiatives while at the same time disguising involvement. For example, McDonald *et al.* (2023) examined the conflict of interest statements provided in publications by researchers who had received funding either directly or indirectly from the tobacco industry.^20^ They found that two-thirds of authors either provided incomplete or no conflicts of interest declarations, highlighting the need to improve processes around conflict of interest declarations. Similarly, Goldner and McCambridge (2021) examined the authorship of 60 systematic reviews into the impacts of alcohol on cardiovascular disease.^21^ They found 14 of these reviews had been undertaken by authors with histories of alcohol funding, all of whom looked closely at the cardioprotective effects of alcohol. Cassidy (2014) conducted in-depth interviews with 49 gambling researchers who talked of the way influence in research is ‘concentrated in the hands of a few individuals who are trusted by industry, regulators and governments to produce research that is acceptable to all parties’ (p. 347).^22^

This willingness to form risky relationships with industry funding is not confined to researchers. Funders and other peak bodies associated with public health research have demonstrated similar morally questionable relationships when it comes to funding from unhealthy commodity industries. For example, in 2014, senior officials at the National Institute on Alcohol Abuse and Alcoholism (NIAAA) actively and secretly courted the alcohol industry to fund a $100 million research trial on ‘Moderate Alcohol and Cardiovascular Health’.^23^ The industry-friendly choice of outcomes contributed to distortions in the science involved. Although exposures by the *New York Times* later led the US National Institutes of Health to force abandonment of the trial, the willingness of NIAAA leadership to engage in such ethically questionable relationships highlights a poor appreciation of moral jeopardy issues.

A likely contributor to public health researchers engaging in relationships of moral jeopardy is a tendency to view them narrowly as focused involvements without seeing how they are impacting more broadly. This blinkered perspective can happen in a variety of ways. First is a tendency to see the immediate advantages of an industry-funded project without taking into account broader implications. For instance, a nutrition researcher might see funds from a sugar-sweetened beverage corporation as an opportunity to improve the product, without appreciating how publication will be used to improve the corporation’s reputation with the public and policymakers.^24^ A second form of narrowing involves thinking of conflicts of interest only in terms of financial arrangements, without realizing that conflicts of interest can function at different levels that include interpersonal and systems dynamics. For example, some think of conflicts of interest only in terms of personal financial conflicts and exclude organizational roles^25^ and others disregard their involvement in industry-supported forums such as conferences and think tanks.^26^ A third form of narrowing involves viewing the transfer of funds as having a cleansing function. For example, a public health nutrition researcher might consider food industry money as ‘clean’ once it is passed on to someone else, perhaps to a PhD student to attend a conference.

In the interests of supporting a wider perspective on moral jeopardy, Table 1 provides a list of the different types of risk that could be considered.

**Table 1.** Moral jeopardy risks associated with receiving funding from industry sources

Type of risk	Description	Example
Ethical	Accepting funds to do good from a source that does harm	A university accepts alcohol company funding for a chair in emergency medicine
Contributory	Accepting funds for projects that contribute to funder’s sales	People are more likely to buy a lottery ticket when some of the profits are put into university breast cancer research
Reputational	Accepting funds is perceived negatively by surrounding stakeholders	A nutrition researcher is shunned by colleagues for exercise research funded by Coca-Cola
Governance	Owner’s independence compromised by reliance on funding	A health promotion agency less likely to speak out about vaping when funding from vaping companies increases from 2% to 10%
Neutrality	Sense of mutual obligation and pro-funder bias that emerges from a period of unhealthy commodity industry funding	A researcher receiving ongoing funding from a petrol automobile manufacturer sees the product in progressively more benign ways
Relationship	Negative impacts of funding on either internal or external relationships	A cancer group accepting alcohol funding weakens health advocacy in adjacent addiction research group
Democratic	Longer-term impacts on democratic systems	A researcher funded by the gun industry plays an active role in supporting the National Rifle Association

Arguably, ‘ethical’ and ‘reputational’ risks are easier to identify and hence more likely to be discussed, but the other types of risk can have diffuse and powerful effects. For example, with regard to ‘governance’ risk, members of a nutrition research group receiving ongoing funding from the beef industry are increasingly more likely to avoid topics that expose the negative impacts of meat on health and senior team members may actively discourage junior team members from communicating in ways that might offend their funders. With regard to ‘relationship’ risks, members of an alcohol research group may feel uncomfortable with their leader accepting industry funding and subsequently decide to resign, resulting in the group losing some of its most valued staff. Each of these possibilities contributes to the gradual erosion of the credibility of the research that is produced.

## Minimizing moral jeopardy

Given the potential of moral jeopardy to undermine the integrity of public health research, what might be done to minimize its impacts? Before addressing this question, we first need to consider who might take responsibility for managing these risks. Individual researchers have a role because it is often down to them to decide on whether to accept industry funding. Research organizations (e.g. universities, health faculties and research institutes) have a role in specifying under what circumstances funding from industries is acceptable or not. The wider community of public health researchers have a role in defining ethical standards and developing systems for improving transparency. Journal editors have a role in specifying expectations and in proactively managing disclosures of conflicts of interest. Governments have a role in devising processes that reduce the influence of industries in distorting the knowledge base. Accordingly, the issue of who is responsible is best seen as a shared and multileveled undertaking.

The following briefly considers three approaches, each operating at different levels and each aiming to minimize moral jeopardy and prevent risky conflicts of interest.

### A continuum of risk approach

Binary ways of looking at the world involve slotting key aspects of what surrounds us into category pairs: the weather is either fine or wet, people are either good or bad, patients are either compliant or disruptive. Thinking in binary categories is tempting and powerful. Their use helps organize a complex picture and facilitates decisive action, but it also runs the risk of oversimplifying and stereotyping what is going on. For example, a common binary related to alcohol is the division of drinkers into those who drink ‘socially’ and those who drink ‘dependently’. The distinction helps to identify people who require assistance, but it also runs the risk of stigmatizing dependent drinkers as weak and qualitatively different from those who drink in other ways. The consequent negative stigma discourages people from talking things over, making them less likely to reflect on their vulnerabilities.

An alternative continuum interpretation involves looking at the world less in terms of fixed categories and more in terms of gradations of intensity across a particular dimension. As with other moral binaries—such as fat/normal weight, alcoholic/social drinker, mad/sane and weak/resilient—the shift from thinking in terms of binaries towards positioning oneself more along a continuum of risk legitimizes the activities of self-reflection and promotes discussion as a normal and legitimate activity.^27^

The key disadvantage of binary interpretations of morality concerns the danger of over-categorizing people into being either willing or not willing to accept profits from industries with health consequences: either they are morally sound or they are not, either they are good or they are corrupt. Such disapproval may contribute to encouraging organizations to reject such funding but, in the longer run, it can also result in people backing away from both deliberation and discussion. The issues become too fraught and raising the topic can be seen as tantamount to accusing someone of ethical cowardice. For example, a government funder who questions a research group regarding whether they receive industry contributions is likely, in a binary world, to encounter a wall of justifications or hostile denials, either of which would discourage further conversation. The concept of a continuum of moral jeopardy avoids these pitfalls. Instead of distancing those who are ambivalent, it recognizes that the extent to which someone engages with industry funding can vary and that it is less a matter of whether or not to accept such funding and more a question of at what point someone deems the relationship to be too risky.

### A self-assessment approach

This approach seeks to create opportunities to discuss, debate and deliberate on the types and intensities of moral jeopardy. One example of this is Odierna’s ‘cycle of bias’ process that engages consumers, healthcare providers and journalists in a series of workshops that help participants appreciate the impacts on research of conflicts of interest and other forms of bias.^28^ In another approach, the first author (P.A.) has developed a procedure called a ‘PERIL Analysis’,^2^^,^^29^ which invites research teams to examine the implications of industry involvement across six indicators, as outlined in Table 2.

**Table 2.** PERIL Analysis indicators

Indicator	Description	Example
**P**urpose	Degree to which purposes between funder and recipient diverge	Direct conflict of purpose occurs when a cancer researcher is funded by a tobacco company
**E**xtent	Degree to which the recipient is reliant on this source	Members of a research team view an alcohol company more positively as funding from them moves from 5% to 10% of their income
**R**elevant harm	Degree of harm associated with this form or subform of consumption	Lottery products are considered to involve less harm than electronic gambling machines
**I**dentifiers	Degree to which the recipient is visibly identified with the funder	A research institute is obliged to display a fast-food company’s logo on all their research reports, seminars and other publications
**L**ink	Nature and directness of the link between recipient and donor	Opioid researchers have a choice of accepting funding directly from a pharmaceutical company or channelled through a government department

Detailed guidelines on how to apply these criteria are laid out in Chapter 14 of *Moral Jeopardy: Risks of Accepting Money from Alcohol, Tobacco and Gambling Industries*^30^ but, to summarize them briefly, members of a research organization (ideally including those in management and on the governance board) put aside time to work through each of the indicators in turn. During their discussion, they attempt to reach a consensus on whether they consider the risks involved to be ‘low’, ‘medium’, ‘high’ or ‘very high’. After finishing their deliberations on all six indicators, they combine these and explore their perceptions of the overall risk. This discussion then informs their collective decision regarding whether to accept this industry’s funding. One option for organizations is to schedule in a PERIL Analysis as a regular part of financial planning and review.

The open discussion of risks helps those present to talk through these sensitive issues without succumbing to accusations or feelings of guilt, then to work towards a collective position. For example, a public health research team considering an offer of funding coming directly from a casino in a publicly visible way is likely to identify high to very high levels of moral jeopardy in terms of ‘purpose’, ‘relevant harm’, ‘identifiers’ and ‘link’ so, in combination, their overall discussion is likely to conclude that this funding is extremely high-risk. However, a health research funder asked to distribute funding from a forestry corporation might consider ‘purpose’ as high-risk but ‘extent’, ‘relevant harm’ and ‘identifiers’ as only moderate-risk, and conclude this is overall only of moderate risk (see also case examples by Thomas Babor).^31–33^

### An ethical positioning approach

The open and visible statement of ethical positions by a particular body achieves several important ends. First, it declares an expectation that is out there, solid and real, and that, in turn, puts the onus on the reader of the message to consider how they are positioned relative to that statement. Second, the statement provides a benchmark for how individuals are expected to behave with one another. Third, it provides a reference point for assessing how individuals or organizations measure up to the stated ideal. This, of course, is double-edged because, once a body declares a position, it, and the people associated with it, will be vulnerable to judgments of how faithfully they adhere to its intentions. Finally, it spells out the legitimacy of speaking out about ethical dilemmas and makes it more permissible for people to speak about ethical issues.

An important example of such an ethical convention is the UN/WHO Framework Convention on Tobacco Control (FCTC) that calls on member governments to adopt a stance of vigilance regarding the varied and dynamic ways in which the tobacco industry is likely to adapt to the way it is constrained (see FCTC, Article 5). Another example is the Committee on Publication Ethics that was set up as a ‘forum for editors and publishers of peer-reviewed journals to discuss all aspects of publication ethics’. It has compiled a set of guidelines on best practice for journal editors that cover many issues including conflicts of interest.

Academic journals can also lead the way in taking ethical positions. At a meeting in Farmington Connecticut in July 1997, representatives of 23 addiction journals agreed on the ‘Farmington Consensus’, which requires members of the subsequently established International Society of Addiction Journal Editors to set ethical standards for authors, referees and editors through measures such as independent review and open declarations of conflicts of interest. Public health journals are also exploring positions on industries with implications for public health. For example, in 2018, the editorial board of the *International Journal of Environmental Research and Public Health*^34^ adopted a new policy of not publishing work that has been funded ‘in whole or part, by a tobacco company or tobacco industry organization or affiliate’ (including e-cigarette funding), nor do they consider papers by authors who accept tobacco industry funding including ‘funding for research costs and for all or part of any author’s salary, or other forms of personal renumeration’ (p. 1). Although these signal stronger recognition of moral jeopardy issues, in practice, implementing such requirements poses some tricky dilemmas. For example, how should an editor respond when, in the declaration of interests section, an author declares ‘none’ when that same author is well known to have engaged previously with industry funding? This highlights the importance for journals of setting up clear procedures for handling conflicts of interest issues.

## Conclusion

With industries with health implications continuing to invest in undermining the evidence base in line with their profit imperatives, the research community can no longer afford to turn a blind eye to their impacts on research integrity. All those involved—funders, research institutes, journal editors and researchers themselves—would benefit from improving their understanding of industry influence and in finding ways of discussing and weighing up the risks of accepting industry funding. Our suggested approaches, such as self-assessment procedures and ethical codes, will help those involved improve their response to moral jeopardy issues in the future. However, although, in principle, these approaches might seem straightforward, in practice, managing conflicts of interest poses significant challenges, particularly in a field in which many involved continue to view industry relationships as unproblematic. As an emergent field, the impacts of different funding arrangements will become clearer as research into the commercial determinants of health gathers momentum.
